# Novel Organotin(IV)-Schiff Base Complexes: Synthesis, Characterization, Antimicrobial Activity, and DNA Interaction Studies

**DOI:** 10.1155/2010/854514

**Published:** 2010-12-30

**Authors:** K. Shiva Prasad, L. Shiva Kumar, Melvin Prasad, Hosakere D. Revanasiddappa

**Affiliations:** ^1^Department of Chemistry, University of Mysore, Manasagangotri, Mysore 570 006, Karnataka, India; ^2^Department of Biotechnology, University of Mysore, Manasagangotri, Mysore 570 006, Karnataka, India

## Abstract

Four organotin(IV) complexes with 2-(2-hydroxybenzylideneamino)isoindoline-1,3-dione (L_1_), and 4-(4-hydroxy-3-methoxybenzylideneamino-*N*-(pyrimidin-2-yl)benzenesulfonamide (L_2_) were synthesized and well characterized by analytical and spectral studies. The synthesized compounds were tested for antimicrobial activity by disc diffusion method. The DNA binding of the complexes **1** and **3** with CT-DNA has been performed with absorption spectroscopy, which showed that both the complexes are avid binders of CT-DNA. Also the nuclease activity of complexes **1** and **3** with plasmid DNA (pUC19) was studied using agarose gel electrophoresis. The complex **1** can act as effective DNA cleaving agent when compared to complex **3** resulting in the nicked form of DNA under physiological conditions. The gel was run both in the absence and presence of the oxidizing agent.

## 1. Introduction

Organotin(IV) complexes have been the subject of interest for some time because of their biomedical and commercial applications [1]. It has been observed that several organotin complexes are effective antifouling, antimicrobial [2], and antiviral agents. The booming application of metal complexes in the treatment of numerous human diseases is a vigorously expanding area in biomedical and inorganic chemistry [3, 4]. The variation in coordination number, geometries, accessible redox states, thermodynamic, and kinetic characteristics and the intrinsic properties of the metal ion are some special characteristics of organometallic complexes that offer the medicinal chemists to employ different strategies for their exploitation. 

 Their use in cancer chemotherapy is gaining mounting importance. Complexes of platinum(II) like cisplatin, oxaliplatin, nedaplatin, and carboplatin have achieved clinical status as a result of intensive research focused on anticancer metal based drugs. However, in spite of having reasonable therapeutic index, the applications of metal complexes are limited by serious disadvantages like (a) poor water solubility and (b) toxicity towards the healthy cells. So the synthesis of nonplatinum chemotherapeutics with positive, no or limited side effects is considered reasonable. Due to the potential antibacterial, antifungal, and anticancer activity, organotins are the subject of intensive investigations [5–8]. In addition to their anti-tumor activities, organotin(IV) complexes with Schiff bases present an interesting variety of structural possibilities [9]. Increasing attention has also been devoted to the organotin(IV) complexes with Schiff base ligands in view of their special anti-tumor activities [10–12]. 

 However, in spite of such an importance the number of reports available on organotin(IV) complexes and their DNA binding properties is very few. As an extension of the studies of organotin(IV) complexes with Schiff bases [13, 14], we have now synthesized four organotin(IV) complexes with Schiff bases, 2-(2-hydroxybenzylideneamino)isoindoline-1,3-dione, and 4-(4-hydroxy-3-methoxybenzylideneamino-*N-*(pyrimidin-2-yl) benzenesulfonamide.

Furthermore, their DNA binding properties and cleavage studies were performed. The objective of the current study is to provide useful insights in the understanding of drug-DNA interaction mechanism and antimicrobial potency of prepared organotin(IV) complexes.

## 2. Experimental

### 2.1. Materials and Reagents

All the chemicals used were of analytical grade and were used without further purification. CT-DNA and pUC19 plasmid DNA were purchased from Sigma. Elemental analyses (carbon, hydrogen, nitrogen, oxygen and sulphur) were performed using a Perkin-Elmer 240 elemental analyzer. IR spectra of the Schiff base ligands and their complexes were recorded on a Shimadzu FT-IR-8300 instrument using KBr discs in the range of 4000–200 cm^−1^. ^1^H-NMR spectra of the synthesized compounds were recorded on Bruker DRX-300 spectrometer using DMSO-d_6_ as solvent and TMS as the internal standard. Molar conductivity measurements were recorded on a CM-82T Elico conductivity bridge in DMSO. Thermogravimetric analyses data were measured from room temperature to 700°C at a heating rate of 20°C/min. The data were recorded on a Shimadzu TG-50 thermobalance. Absorption spectra were recorded using a hp spectrophotometer (Agilent 8453).

### 2.2. Synthesis of Ligands

**Scheme 1:**
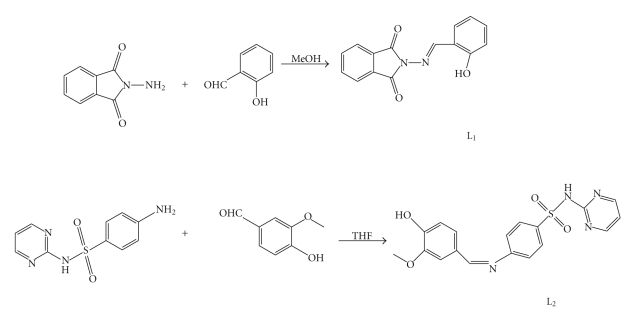
Synthetic route of the ligands.

The condensation of N-aminophthalimide or sulfadiazine with salicylaldehyde was done in methanol (L_1_) and THF (L_2_). A solution of N-aminophthalimide (0.3221 g, 2 mmol) in 15 cm^3^ of methanol or a solution of sulfadiazine (0.500 g, 2 mmol) in 15 cm^3^ of THF was added slowly with constant stirring to a solution of salicylaldehyde (2 mmol) and the reaction mixture was refluxed for 4 hs (Scheme 1). The colorless products obtained were filtered, washed with hot water and dried in air. The products were crystallized from ethanol.

### 2.3. Synthesis of Organotin(IV) Complexes

A methanolic solution (15 cm^3^) of the above prepared two ligands (1 mmol) was allowed separately to react with a methanolic solution of dimethyltin(IV)dichloride (0.221 g, 1 mmol) species in 1 : 1 and 1 : 1:  1 using methanolic solution of 1,10-phenanthroline (0.198 g, 1 mmol) as another bidentate ligand. The reaction mixture was stirred continuously for 1 h and refluxed for 3 h, respectively, for the preparation of 1 : 1 and 1 : 1:  1 complexes. The precipitate obtained was filtered, washed with hot methanol, and dried in air.

### 2.4. Antimicrobial Activity

The *in vitro* antimicrobial screening effects of the synthesized ligands and their corresponding organotin complexes were evaluated against five bacterial strains, namely, *Bacillus subtilis, E. coli, Staphylococcus aureus, Ralstonia solanacearum,* and *Xanthomonas vesicatoria *by paper disc diffusion method, using nutrient agar medium. The antifungal activities of the compounds were evaluated against *Aspergillus niger, Aspergillus flavus, Fusarium oxysporum *and* Alternaria solani* by disc diffusion technique using potato dextrose agar as medium [15, 16]. All the tests were performed in triplicate, and average is reported.

### 2.5. DNA Binding Studies

The DNA concentration per nucleotide was determined adopting absorption spectroscopy using the known molar extinction coefficient value of 6600 M^−1^ cm^−1^ at 260 nm [17]. Absorption titrations were performed by using a fixed metal complex concentration to which increments of the DNA stock solution were added. Metal complex DNA solutions were incubated for 10 min before the absorption spectra were recorded.

### 2.6. Viscosity Measurements

Viscosity measurements were carried out using an Ubbelodhe viscometer at room temperature. The viscometric studies were carried out in buffer solution (pH 7.2). Flow time was measured by hand with digital stopwatch, each sample was measured three times, and the average flow time was calculated. The data were reported as (*η*/*η*_o_)^ 1/3^ versus the binding ratio [18], where *η* is the viscosity of DNA in the presence of the compound and *η*_o_ is the viscosity of DNA solution alone. Viscosity values were calculated from the observed flow time of DNA containing solution corrected for the flow time of the buffer alone.

### 2.7. Photonuclease Activity

The gel electrophoresis experiments using supercoiled pUC19 DNA were carried out as reported previously [19, 20]. The extent of cleavage was measured from the intensities of the bands using the Alpha Innotech Gel documentation system (AlphaImager 2200). For mechanistic investigation, reactions were carried out by adding radical scavenging agents (H_2_O_2_) to supercoiled DNA prior to the addition of the complex before incubation.

## 3. Results and Discussion

 All the metal complexes are air stable and decompose at higher temperature (>300°C). The complexes are insoluble in water, ethanol, and methanol but soluble in DMF and DMSO. The analytical data along with some physical properties of the ligands and their metal complexes are reported in Table 1. The molar conductivity values of complexes in DMSO are in the range of 4.25–9.22 S cm^2^ mol^−1^ indicating their nonelectrolytic nature [21].

## 4. Infrared Spectra

The IR spectra of the free ligands were compared with the spectra of the organotin(IV) complexes in order to study the binding mode of Schiff bases to organotin(IV) in the new complexes. Several significant changes with respect to the ligands are observed in the corresponding diorganotin(IV) complexes, which are listed in Table 2. A sharp band at 1725 cm^−1^ in L_1_ is due to *ν*(C=O) is completely merged in complex **1** indicating the chelation of the ligand moiety to tin with the oxygen atom and in complex **2** it is found to be at the same position suggesting the noninvolvement of the carbonyl group of L_1_ with the central atom. The IR band of another carbonyl group is slightly affected by bending and because of this bending the band at 1768 cm^−1^ due to *ν*(C=O) in L_1_ decreased to 1676 cm^−1^ in complex **1** and to 1650 cm^−1^ in complex **2**. A sharp band at 3384 cm^−1^ in L_1_ due to *ν*(–OH) is found to be disappearing in both the complexes **1** and **2**, indicating the deprotonated phenolic oxygen donor site to the tin atom. It was also found that the band at 1618 cm^−1^ in L_1_ due to *ν*(C=N) is absent in the complex **1** and it was slightly shifted to 1617 cm^−1^ in the complex **2** indicating the involvement of N-atom of azomethine group in the coordination with the tin. Comparing the IR spectra of L_2_ with those of complex of **3** and **4** gave important information. A characteristic band at 1652 cm^−1^ in L_2_ due to *ν*(C=N) is found to be disappearing in the complex **3,** and it was shifted to 1658 cm^−1^ in complex **4** indicating the chelation of the N-atom of the ligand moiety to the tin atom. The band at 3423 cm^−1^ due to *ν*(–OH) is found to be disappearing in the case of complex **3** indicating the chelation of oxygen atom to the tin. In the complex **4,** it is found unchanged indicating the noninvolvement of the phenolic oxygen in the chelation with the central tin atom. The characteristic band at 941 cm^−1^ in L_2_ due to *ν*(–N–SO_2_) is shifted to 891 cm^−1^ in the complex **3** indicating the involvement of N-atom in the complexation, and it was found that the peak position was unchanged in case of complex **4** indicating the non-involvement of N-atom in the coordination process. Similarly, the band at 2360 cm^−1^ due to *ν*(–NH) is shifted to 2399 cm^−1^ in the complex **3** indicating that the N-atom is involved in coordination with tin atom and also suggesting the structure of complex **3** polymeric in nature, and it was found that the peak was remained in the same position indicating that the N-atom is not involved in the coordination.

## 5. ^**1**^H-NMR Spectra

The bonding pattern is further supported by ^1^H-NMR spectral studies of ligands and their corresponding tin complexes. The signal at 9.45 ppm in L_1_ and at 9.77 ppm in L_2_ is assigned to azomethine (HC=N) proton, which was shifted to down field in the spectra of all the complexes; this is attributed to the donation of the lone pair of electrons by the azomethine nitrogen to the tin atom. The –OH proton signal in L_1_ at 10.67 ppm was found to be shifted to 10.63–10.65 ppm indicating the involvement of phenolic oxygen in the coordination in complexes **1** and **2**. In L_2_ the –OH signal was at 10.26 ppm and is shifted to 10.28 ppm in the complex **3 **and remained unchanged in complex **4**, indicating that the oxygen atom is involved in the chelation with the central tin atom in complex **3**. The ^1^H-NMR spectra of ligands in DMSO-d_6_ revealed a multiplet at 6.01–7.95 ppm corresponding to aromatic protons [22]. All the complexes show signal around 1.00–1.03 ppm, which corresponds to Sn–CH_3_ group. ^1^H-NMR data of ligands and their complexes are presented in Table 3.

## 6. Electronic Spectra

 Electronic spectra of the ligands and their metal complexes were recorded in DMF. An absorption band found at 351 nm in L_1_ is due to *n* → π* transition, and was decreased in its complex **1** to 344 nm having *n* → π* transition, and complex **2** has another band found at 384 nm, having ligand field transition. A band at 340 nm in L_2_ has been found at 340 nm in complex **3 **having *n* → π* transition and at 380 nm in complex **4** having ligand field transition. The spectra of the complexes are dominated by intense intraligand and charge transfer bands, since it is known that metal/metalloids are capable of forming d*π*-p*π* bonds with ligands containing nitrogen and oxygen as donor atoms [23].

## 7. Thermal Studies

**Figure 1:**
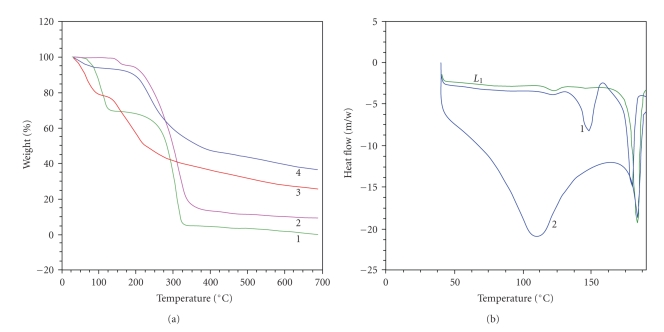
(a) Thermogravimetric (TGA) curve of organotin(IV) complexes (**1–4**) and (b) DSC curve of ligand (L_1_) and complexes (**1** and **2**).

The thermal analysis of the ligand (L_1_) and all the synthesized organotin(IV) complexes was carried out under an inert atmosphere (N_2_). The ligand (L_1_) exhibits one-step thermolytic pattern while all the complexes decompose in three stages (Figures 1(a) and 1(b)). The decomposition of the free ligand begins at 156°C and exhibits a sharp endothermic peak (absent in the complexes **1** and **2**) at 183°C. An examination of the TG curves of the complexes indicates that decomposition runs in three steps at 105–115°C, 115–185°C, and 185–360°C. Afterwards the complexes decompose continuously up to 700°C. The first and second steps are suggested to represent expulsion of two methyl groups, *calc. *(7.2%, 5.0%, 7.8%, and 4.2% for the complexes **1, 2, 3,** and **4, **resp.), found (7.7%, 5.4%, 8.2% and 4.4% for complexes **1, 2, 3,** and **4, **resp.). In the second step, decomposition of ligand moiety was observed as *calc. *(64%, 78.1%, 42.0%, and 81.6%), found (64.3%, 78.7%, 42.3%, and 82.0%), for complexes **1**–**4**, respectively. Third stage of pyrolysis corresponds to 21.3%, 19.8%, 12.9% and 16.5% weight losses for the complexes **1**, **2, 3,** and **4,** respectively, and was consistent with the expulsion of phenanthroline moiety leaving behind metal oxide as the end-product. 

 The DSC plots are consistent with the decomposition pattern of the ligand (L_1_) and its complexes (**1** and **2**). A peak observed at 184°C indicates the pyrolysis of the ligand as an exothermic process. However, there is no well-defined peak for the formation of metal oxide as the end product.

**Figure 2:**
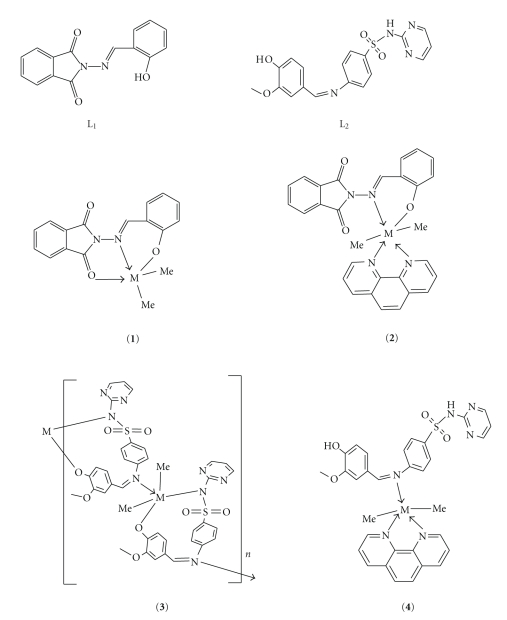
Structures of ligands and organotin(IV) complexes [M=Sn(IV)].

Based on these, the following structures are assigned to the prepared complexes (Figure 2).

## 8. Biological Results

### 8.1. Antimicrobial Activity

*In vitro* antimicrobial activity of the ligands and their corresponding organotin complexes was tested by the paper disc diffusion method [24, 25] at 200 *μ*g/mL concentration in DMF. The bacteria were subcultured in agar medium, and the Petri dishes were incubated for 24 h at 37°C. Standard antibacterial drug (Chloramphenicol) was also screened under similar conditions for comparison. The fungi were subcultured in potato dextrose agar medium and the Petri dishes were incubated for 72 hs at 37°C. Standard antifungal drug (Griseofulvin) was used for comparison. The wells were dug into the agar media using a sterile metallic borer. Activity was determined by measuring the diameter of the zone showing complete inhibition (mm). Growth inhibition was compared with the standard drugs (positive drug). Negative controls were prepared using the same solvent, DMF, employed to dissolve the test compounds (no activity against any microbial strains). By the observation of zones of inhibition, it was concluded that the organotin complexes are more active than free ligands, which indicates that the metallation increases antimicrobial activity. The above studies reveal that the organotin complexes synthesized in the present work are highly active against all the selected microorganisms. The results reported in Table 4 reveal that all the organotin complexes are particularly active against bacteria *E. coli, S. aureus, R. solanacearum *and against fungi, namely,* A. niger *and* A. solani. *The complexes showed moderate activity against other selected species of microorganisms. 

### 8.2. DNA Binding Experiments

DNA binding experiments for the complexes were performed in Tris-HCl/NaCl buffer (5 mM Tris-HCl, 5 mM NaCl, pH 7.2) using aqueous solution of the complexes. Calf-thymus DNA (CT-DNA) in Tris-HCl buffer gave a ratio of the UV absorbance at 260 and 280 nm of *ca* 1.9 : 1 indicating the purity of DNA which is apparently free from proteins [26]. The concentration of DNA was calculated from its absorption intensity at 260 nm with known molar absorption coefficient value of 6600 cm^−1^. UV-Vis absorption titration experiments were performed by varying the concentration of CT-DNA keeping the metal complex concentration constant (50 *μ*M). Samples were allowed to get equilibrated to bind sufficiently to CT-DNA before recording each spectrum. From the observed data, the intrinsic binding constant, *K_b_* was calculated by using the following [27]:

(1) $$\frac{\left[\text{DNA}\right]}{\left(\varepsilon_{a}-\varepsilon_{f}\right)}=\frac{\left[\text{DNA}\right]}{\left(\varepsilon_{b}-\varepsilon_{f}\right)}+\frac{1}{K_{b}}\left(\varepsilon_{a}-\varepsilon_{f}\right),$$

where *ε*_*a*_, *ε*_*f*_, *ε*_*b*_ are the apparent, free, and bound metal complex extinction coefficients. A plot of [DNA]/(*ε*_*a*_ − *ε*_*f*_) versus [DNA] gave a slope of 1/(*ε*_*b*_ − *ε*_*f*_) and a “*y*” intercept equal to 1/*K_b_*  (*ε*_*a*_ − *ε*_*f*_), where *K_b_* is the ratio of the slope to the *y* intercept.

 Absorption spectra of the complexes **1** and **3** in the absence and in the presence of CT-DNA are shown in Figures 4 and 5. With increasing concentration of CT-DNA, the absorption bands were affected and show minor bathochromic shift of the spectral band of ~4 nm with significant hypochromicity, suggesting mainly groove binding property of complexes to the double-stranded DNA [28]. Both ligands exhibit comparatively less binding propensity to the CT-DNA. The DNA binding property of complex **1** could be due to the presence of indoline system and that of complex **3** could be due to the presence of extended pyrimidine ring system.

### 8.3. Viscosity Measurements

The nature of binding of the complexes **1** and **3** to the CT-DNA was further investigated by viscometric studies. Viscosity measurements were carried out in buffer solution (pH 7.2) using an Ubbelodhe viscometer at room temperature. Flow time was measured by hand with digital stopwatch, each sample was measured three times, and the average flow time was calculated. A significant increase in the viscosity of DNA on addition of complex results due to the intercalation which leads to the separation among the DNA bases in the increase in the effective size in DNA which could be the reason for the increase in the viscosity [29]. Plot of (*η*/*η*_*o*_)^1/3^ versus [complex]/[DNA] gives a measure of the viscosity changes (Figure 6). A gradual increase in the relative viscosity was observed on addition of the complexes **1** and **3** to DNA solution suggesting mainly groove binding nature of the complexes.

### 8.4. DNA Cleavage

**Figure 3:**
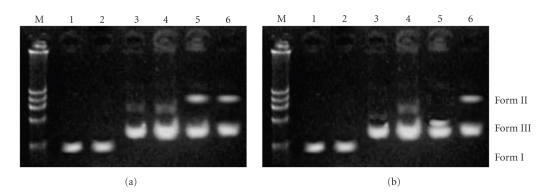
Cleavage of supercoiled pUC19 DNA (0.5 *μ*g) by the ligands and complexes (a) **1 **and (b) **3 **in a buffer containing 50 mM Tris-HCl at 37°C (30 min). lane M: marker; lane 1: DNA control; lane 2: L_1_ (10^−3^ M) + DNA; lane 3: DNA + H_2_O_2_; lane 4: L_2_ + DNA + H_2_O_2_; lane 5: complex (10^−3^ M) + DNA; lane 6: complex + DNA + H_2_O_2_.

**Figure 4:**
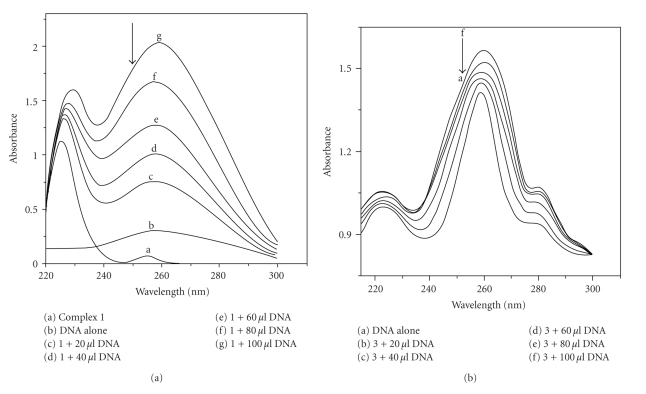
Absorption spectra of (a) complex **1** (5.0 × 10^−5^ M) and (b) complex **3** (5.0 × 10^−5^ M), in Tris-HCl buffer upon addition of DNA = 0.5 *μ*M, 0–100 *μ*L. Arrow shows the absorbance changing upon increasing the concentration of DNA.

**Figure 5:**
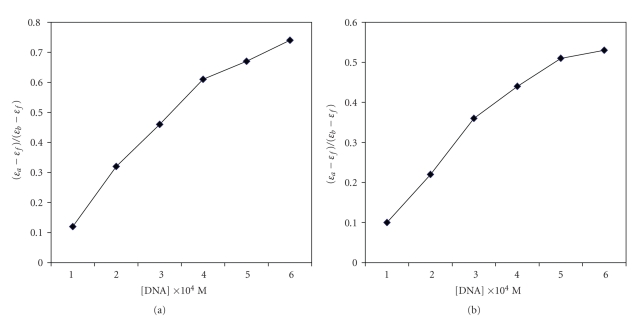
The plot of [DNA]/(*ε*_*a*_ − *ε*_*f*_) versus [DNA] for the titration of DNA with the complexes (a) **1** and (b) **3**.

**Figure 6:**
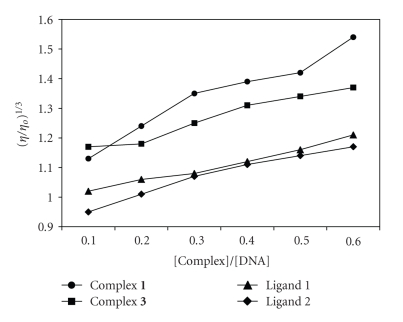
Effect of increasing amounts of complexes (**1 **and **3**) and ligands on the relative viscosity of CT-DNA at 25 ± 0.1°C.

The DNA cleavage experiments of supercoiled (SC) pUC19 DNA by the organotin(IV) complexes was studied by agarose gel electrophoresis. DMF solutions of the complexes were placed in clean eppendrrof tubes, and 1 *μ*g of pUC19 DNA was added. The contents were incubated for 30 min at 37°C and loaded on 0.8% agarose gel after mixing with 5 mL of loading buffer [0.25% bromophenol blue + 0.25% xylene cyanol + 30% glycerol (2 *μ*L) + sterilized distilled water], and the solution was finally loaded on 0.8% agarose gel containing 1.0 *μ*g/mL ethidium bromide (EB). Electrophoresis was performed at constant voltage (70 V) until the bromophenol blue reached to the 3/4th of the gel. The bands were visualized by UV light and photographed. The extent of cleavage of SC DNA was determined by the ability of complex to form open circular (OC) or nicked circular (NC) DNA from its supercoiled (SC) form. The extent of DNA cleavage, observed by agarose gel electrophoresis, gives the order 1>3≫4>2 (Figures 3(a) and 3(b)).

## 9. Conclusion

In conclusion, we have synthesized two important Schiff base ligands and their organotin(IV) complexes. The structures of synthesized ligands and complexes were confirmed by the analytical, spectral, and thermal studies. Antimicrobial results reveal that activity enhances upon coordination, which were compared with standard drugs. All the organotin complexes are particularly active against bacteria *E. coli, S. aureus, R. solanacearum *and against fungi, *namely*, * A. niger *and* A. solani. * The interaction of complexes **1** and **3 **with CT-DNA has been investigated with UV spectroscopic, and viscosity studies have shown that the complexes are simple groove binders to the CT-DNA. The photocleavage studies of complexes **1** and **3** were performed, and results reveal that both the complexes are capable of promoting the cleavage of plasmid DNA under physiological conditions.

## Figures and Tables

**Table 1:** Analytical data and some physical properties of the Schiff base ligands and their organotin(IV) complexes.

Compound	Mol. formula	Yield%	*ω* *_i_* (calc.)/%					Molar conductivity Scm^2^ mol^−1^
			*ω* *_i_* (found)/%					
			C	H	S	O	N	
L_1_	C_15_H_10_N_2_O_3_	84	67.41 (67.60)	3.72 (3.75)	—	17.88 (18.02)	10.32 (10.51)	—
**1**	C_17_H_15_N_2_O_3_Sn	67	49.09 (49.32)	3.51 (3.65)	—	11.42 (11.59)	6.66 (6.76)	9.20
**2**	C_29_H_23_N_4_O_3_Sn	73	58.52 (58.61)	3.78 (3.90)	—	7.82 (8.02)	9.31 (9.42)	4.25
L_2_	C_18_H_16_N_4_O_4_S	82	55.91 (56.24)	4.01 (4.20)	8.19 (8.34)	16.3 (16.65)	14.41 (1457)	—
**3**	C_38_H_34_N_8_O_8_S_2_Sn	57	49.86 (49.95)	3.61 (3.75)	6.91 (7.01)	13.87 (14.01)	12.09 (12.26)	5.62
**4**	C_32_H_30_N_6_O_4_SSn	62	52.11 (53.87)	4.91 (4.23)	4.28 (4.49)	8.66 (8.97)	11.61 (11.78)	8.93

**Table 2:** Infrared spectral data of the ligands and their organotin(IV) complexes.

	***ν*/**cm^−1^					
compound	***ν***(C=O)	***ν***(O–H)	***ν***(C=N)	***ν***(M–N)	***ν***(M–O)	***ν***(–N–SO_2_)
L_1_	1768 and 1725	3384	1618	—	—	—
**1**	1676	—	—	512	384	—
**2**	1650 and 1725	—	1617	516	417	—
L_2_	—	3423	1652	—	—	941
**3**	—	—	—	480 and 461	403	891
**4**	—	3423	1658	415	—	941

**Table 3:** ^1^H-NMR data of the ligands and organotin(IV) complexes.

compound	HC=N	–OH	Ar–H	–NH	Sn–CH_3_	O–CH_3_
L_1_	9.45	10.67	6.92–7.91	—	—	—
**1**	9.46	10.63	6.90–7.88	—	—	—
**2**	9.46	10.65	6.92–7.94	—	—	—
L_2_	9.77	10.26	6.01–7.95	11.24	—	3.84
**3**	9.77	10.28	6.01–7.93	11.30	1.03	3.84
**4**	9.75	10.26	6.01–7.95	—	1.03	3.82

**Table 4:** Antimicrobial activity of Schiff bases and their corresponding organotin(IV) complexes.

Compound	Antibacterial activity					Antifungal activity			
	Zone of inhibition (in mm)*								
	*B. subtilis*	*E. coli*	*S. aureus*	*R. solanacearum*	*X. vesicatoria*	*A. niger*	*A. flavus*	*F. oxysporum*	*A. solani*
L_1_	05	04	06	08	04	07	05	04	08
**1**	20	23	21	27	22	24	21	19	22
**2**	17	24	19	23	19	22	17	18	21
L_2_	09	06	05	11	04	09	08	05	10
**3**	19	11	20	26	21	23	20	21	23
**4**	14	12	17	21	15	21	15	17	19
Chloramphenicol	29	26	25	32	35	—	—	—	—
Griseofulvin	—	—	—	—	—	27	23	26	25

*Average of three replicates.
